# Characterization of activity behavior using a digital medicine system and comparison to medication ingestion in patients with serious mental illness

**DOI:** 10.1038/s41746-021-00436-1

**Published:** 2021-04-06

**Authors:** Jeffrey M. Cochran, Zahra Heidary, Jonathan Knights

**Affiliations:** Otsuka Pharmaceutical Development & Commercialization, Inc., Princeton, NJ USA

**Keywords:** Outcomes research, Translational research

## Abstract

Activity patterns can be important indicators in patients with serious mental illness. Here, we utilized an accelerometer and electrocardiogram incorporated within a digital medicine system, which also provides objective medication ingestion records, to explore markers of patient activity and investigate whether these markers of behavioral change are related to medication adherence. We developed an activity rhythm score to measure the consistency of step count patterns across the treatment regimen and explored the intensity of activity during active intervals. We then compared these activity features to ingestion behavior, both on a daily basis, using daily features and single-day ingestion behavior, and at the patient-level, using aggregate features and overall ingestion rates. Higher values of the single-day features for both the activity rhythm and activity intensity scores were associated with higher rates of ingestion on the following day. Patients with a mean activity rhythm score greater than the patient-level median were also shown to have higher overall ingestion rates than patients with lower activity rhythm scores (*p* = 0.004). These initial insights demonstrate the ability of digital medicine to enable the development of digital behavioral markers that can be compared to previously unavailable objective ingestion information to improve medication adherence.

## Introduction

Activity patterns and circadian rhythm are often disrupted in patients with serious mental illness (SMI)^1–5^, and thus, characterizing related behaviors could provide useful behavioral markers that enable better understanding and assessment of patients’ disease state. Wearable sensor data, such as accelerometer-derived step count and electrocardiogram- (ECG-) measured heart rate, have been used to quantify this activity markers^6–9^.

Additionally, insufficient medication adherence is a significant concern for patients with serious mental illness^10,11^ and can lead to poor outcomes, such as the increased risk of relapse and diminished quality of life^12,13^, as well as increased utilization of the healthcare system^10,14^. Traditional methods of inferring medication adherence are typically subjective or only track proxy markers of ingestion^15,16^. This limitation presents a clear opportunity for digital medicine, which refers to the combination of an active pharmaceutical with an ingestible sensor that enables the objective recording of medication ingestion via a mobile application^17^, providing more direct insight into patients’ medication ingestion patterns^18,19^ than other common proxy measures of adherence.

**Fig. 1:**
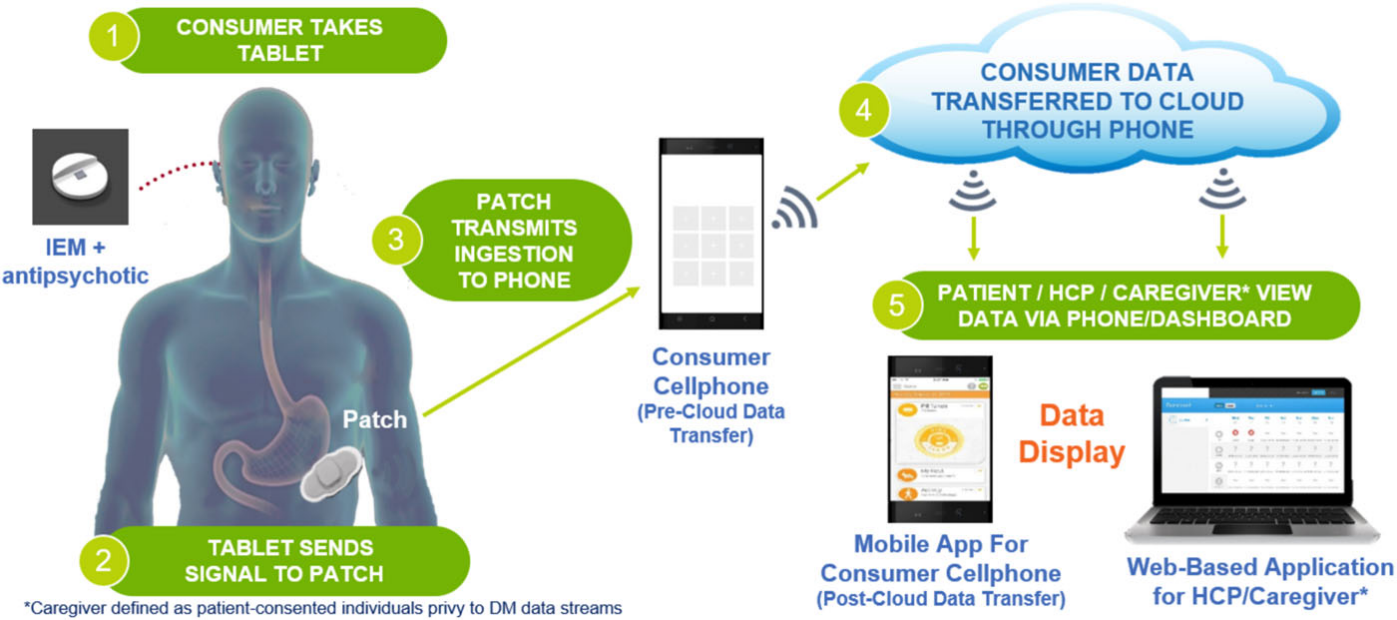
Schematic of the digital medicine system (DMS). The DMS consists of an ingestible sensor embedded within a tablet of aripiprazole. The sensor logs an ingestion by communicating with a torso-worn patch. In addition to recording ingestion events, the patch also contains an accelerometer and ECG. Data are sent from the patch to a smartphone app and stored on a cloud server where it can be accessed by patients or designated caregivers.

The digital medicine system (DMS) utilized here^17^ also non-invasively records complementary information, such as step count and heart rate, that can provide further insight into patient behavior (Fig. 1). The combination of this behavioral data with objective ingestion data provides a unique opportunity to explore relationships between patterns of patient activity and medication ingestion behavior, which could both contextualize the behaviors that are associated with good or poor adherence and lead to the development of behavioral markers of adherence that could be more broadly applied.

Here, we present a retrospective analysis of the activity cycles of patients with serious mental illness using the DMS. First, we analyzed the consistency of subjects’ step count patterns by comparing the most prominent spectral feature in each subject’s step count oscillations across their treatment regimens. We also explored subjects’ heart rates during active intervals compared to their mean daily heart rates as a potential marker of activity intensity. In order to explore whether these behavioral markers were related to differences in patient ingestion patterns, the activity metrics were compared to medication ingestion at both a patient-level and a daily level. Highly consistent activity patterns were linked to higher rates of medication ingestion.

## Results

### DMS data

In this analysis, accelerometer-based step counts, measured at 1-min intervals, and ECG-derived mean heart rates, measured at 5-min intervals, were used to characterize patient activity patterns. These data were then partitioned into 15-min intervals, with the total step count and mean heart rate calculated across each interval. Additionally, time-stamped ingestion records were recorded when a patient wearing the DMS patch ingested a pill.

### Activity rhythm feature

**Fig. 2:**
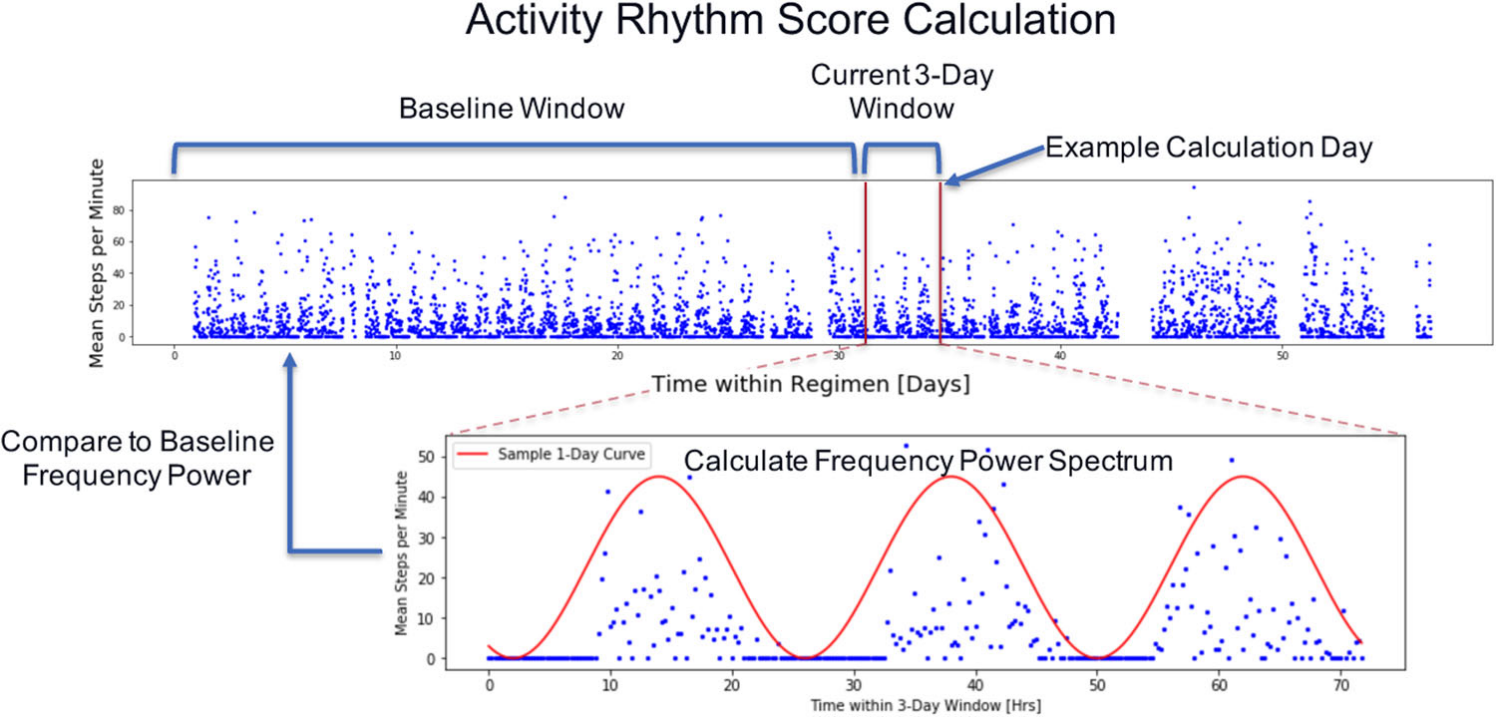
Activity rhythm (*AR*) score calculation example. In order to calculate *AR* for a given day, the Lomb–Scargle periodogram is applied to a 3-day of window of step-count data leading up to and including that day, and a characteristic frequency *f*_c_ is calculated for the time-series. *AR* is then defined as the amplitude of that Lomb–Scargle periodogram at the mean of all characteristic frequencies from earlier days divided by the amplitude of the periodogram at *f*_c_. Thus, *AR* is a measure of how consistent the step count pattern is, relative to the pattern of earlier days.

In order to characterize the consistency of activity patterns, the time-series of the 15-min resolution step count data for a given day and the two preceding days was analyzed using the Lomb–Scargle periodogram^20–22^, which produced a power spectral density (*P*) and characteristic frequency (*f*_c_) for each day that had a sufficient amount of data (see “Methods” section). Because we were interested in the consistency of a patient’s step count relative to previous days’ patterns, we then defined an activity rhythm score (*AR*), such that

1 $$AR_m = \frac{{P_m\left( {\left\langle f \right\rangle } \right)}}{{P_m\left( {f_{\mathrm{c}}^m} \right)}},$$

where *AR*_*m*_ is the activity rhythm score on the *m*th day, $f_{\mathrm{c}}^m$ is the characteristic frequency on the *m*th day, *P*_*m*_ is the Lomb–Scargle periodogram amplitude calculated using the 3-day window of step count data ending on the *m*th day, and 〈*f*〉 is the mean of the characteristic frequencies of all previous days for that patient (Fig. 2). By this definition, larger values of *AR*_*m*_ indicate that the *m*th day step count pattern is more consistent with previous days’ activity rhythms.

### Active heart-rate feature

**Fig. 3:**
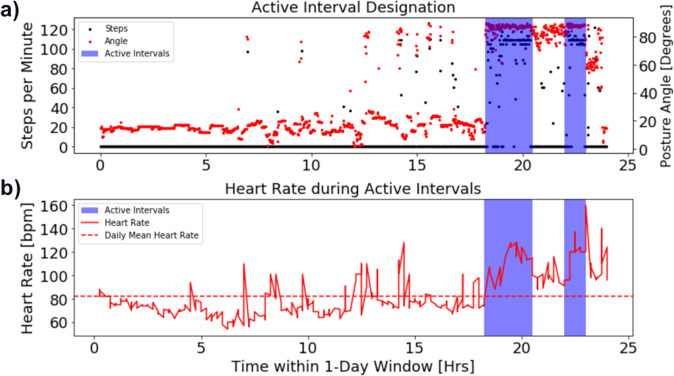
Active-interval relative heart rate calculation example. **a** For each patient-day, active intervals are defined as those 15-min intervals in which at least one-third of the interval’s accelerometer records contained a posture angle greater than or equal to 30° and a step count greater than 0. **b** The mean heart rate was then calculated within the active intervals and normalized to the overall mean heart rate for that day to calculate the active-interval relative heart rate *rHR*_*m*_.

In order to characterize the intensity of activity undertaken by patients, we also identified active 15-min intervals for each patient-day, by classifying as “active” any interval in which at least one-third of the available accelerometer records within the 15-min interval reported a vertical posture angle, i.e., an angle greater than or equal to 30° above horizontal, and a step count greater than 0 (Fig. 3a). We then calculated an active-interval relative heart rate *rHR*_*A*_, such that

2 $$rHR_m = \frac{{\left\langle {Active\,HR} \right\rangle _m}}{{\left\langle {HR} \right\rangle _m}},$$

where *rHR*_*m*_ is the patient’s active-interval relative heart rate on the *m*th day, 〈*Active HR*〉_*m*_ is the average heart rate within all active intervals on the *m*th day, and 〈*HR*〉_*m*_ is the patient’s average heart rate across the entirety of the *m*th day (Fig. 3b).

### Study population

The population analyzed here included 113 subjects with SMI across two separately conducted clinical trials^23,24^. Table 1 contains a summary of the clinical and demographic features of the subjects in these trials. Of the available population, 95 patients had data that was analyzable per the criteria discussed in the “Methods” section, resulting in a total of 2525 analyzable days, i.e., days on which the *AR* score could be calculated, of a possible 4231 days for which this metric could have been calculated across the full 113-subject data set. The median number of days per patient was 29 with an interquartile range (IQR) of 23.5.

**Table 1:** Demographic and clinical characteristics of sample.

Sample size, *n*	113
Females: males (% female)	47:66 (42%)
Age, years (mean ± st. dev.)	46.5 ± 11.1
Race (%)	
Black or African-American	67 (59%)
White	39 (35%)
Asian	5 (4%)
Other	2 (2%)
Ethnicity	
Hispanic or Latino	5 (4%)
Not Hispanic or Latino	108 (96%)
Diagnosis	
Schizophrenia	79 (70%)
Bipolar 1 disorder	22 (19%)
Major depressive disorder	12 (11%)
Aripiprazole dose	
2 mg	7 (6%)
5 mg	8 (7%)
10 mg	30 (27%)
15 mg	26 (23%)
20 mg	18 (16%)
30 mg	24 (21%)
Number of days on DMS (mean ± st. dev.)	49.9 ± 10.8
Mean daily step count (mean ± st. dev.)	7480 ± 4258
Mean heart rate (mean ± st. dev.)	85 ± 10

### Comparison of activity features and ingestion

**Fig. 4:**
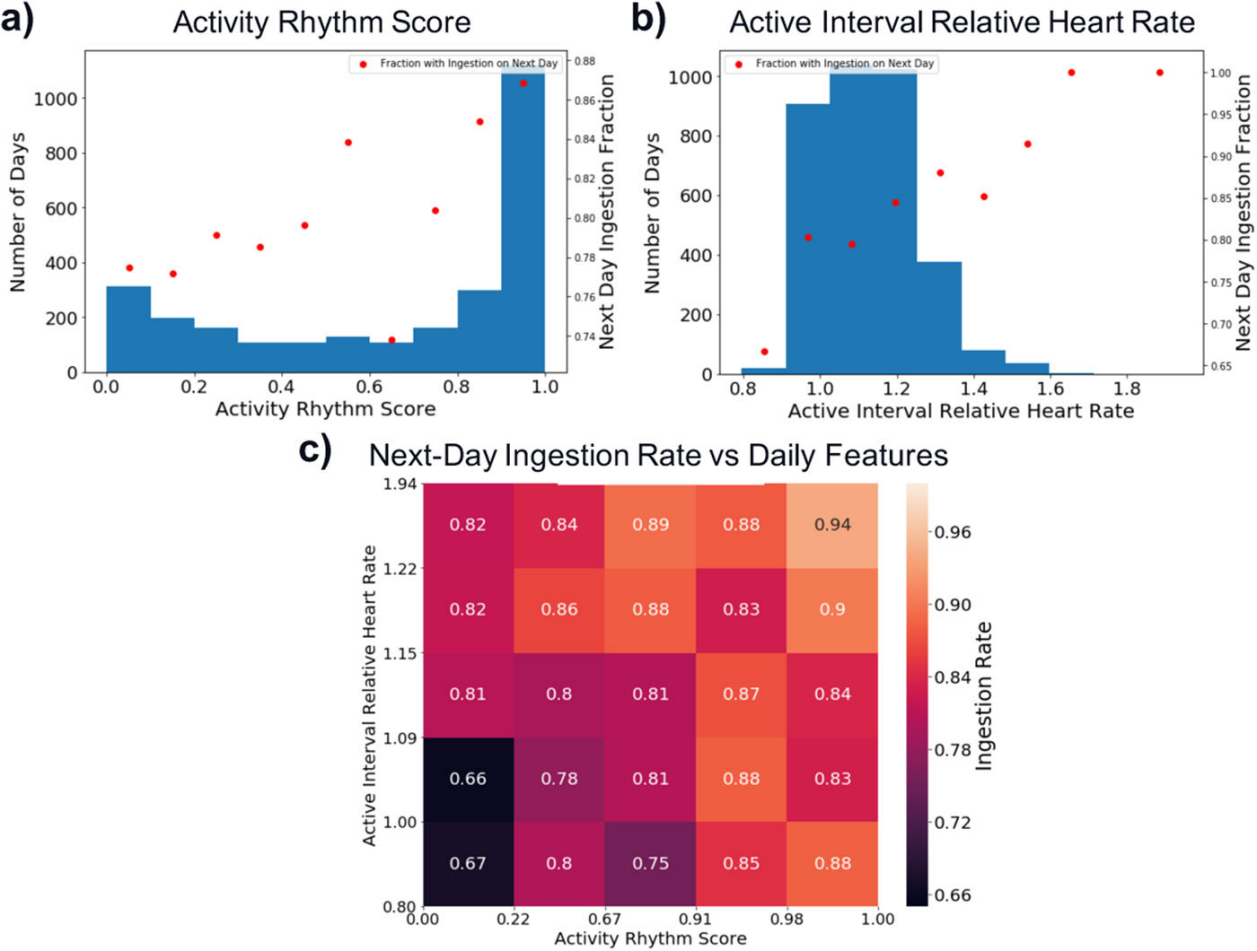
Daily *AR* and *rHR* Features vs next-day ingestion. **a** Histogram of *AR* values for all analyzable days. The red dots represent the fraction of days within each bin that are followed by successful ingestion on the next day. Note that the next-day ingestion rate seems to be higher for days with higher *AR* values. **b** Histogram of *rHR* values for all analyzable days. The red dots represent the fraction of days within each bin that are followed by successful ingestion on the next day. Note that the next-day ingestion rate seems to be higher for days with higher *rHR* values. **c** Heatmap of next-day ingestion rates as a function of both *AR* and *rHR*. The analyzable days are sorted into bins based on their *AR* quintile (*x* axis) and *rHR* quintile (*y* axis), and the color of the heatmap represents the fraction of days in each bin that are followed by successful ingestions on the next day. Note that the values on the *x* and *y* axes represent the limits of each quintile for the features. Higher values of both *AR* and *rHR* seem to be associated with higher next-day ingestion rates.

We first explored the relationship between daily *AR* and *rHR* and next-day ingestion by using all 2525 analyzable days, irrespective of the patient. Of these days, 83% had recorded ingestions on the following day. A summary of the daily activity features can be found in Table 2. Figure 4a, b contains histograms of the activity features, as well as the fraction of days within each histogram bin that was followed by an ingestion on the next day. Higher daily *AR* values are associated with an increased likelihood of an ingestion on the following day. The same relationship seems to hold for next-day ingestion and daily *rHR*. Figure 4c demonstrates this finding across both dimensions with a heatmap for the next-day ingestion as a function of both *AR* and *rHR*.

**Table 2:** Feature summary.

Feature	Median (interquartile range)
Daily features	
Next-day ingestion rate—all days	0.83
*AR*	0.81 (0.68)
*rHR*	1.12 (0.19)
Patient-level features	
Overall ingestion rate	0.88 (0.16)
Number of analyzable days	29.0 (23.5)
Fraction of DMS days that are analyzable	0.53 (0.50)
〈*AR*〉	0.75 (0.60)
*σ*[*AR*]	0.20 (0.16)
〈*rHR*〉	1.14 (0.08)
*σ*[*rHR*]	0.09 (0.06)

Daily features across all patients are presented. Patient-level mean and standard deviation values are calculated across all analyzable days for each patient. The medians and interquartile ranges for these features are presented here.

**Fig. 5:**
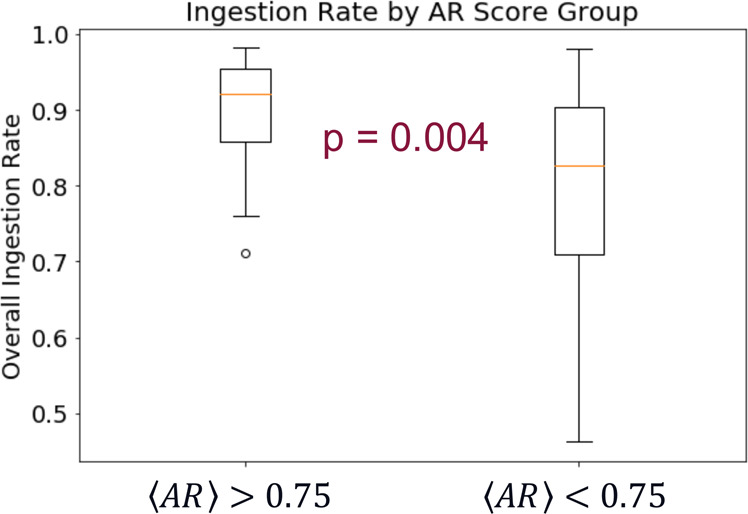
Patient-level *AR* versus overall ingestion rate. Boxplot of the overall ingestion rate for these patients divided at the median 〈*AR*〉 value of 0.75 into high- and low-〈*AR*〉 groups. Here, the center lines of the boxplots represent the median value, the box limits represent the upper and lower quartiles, the whiskers extend to 1.5 times the interquartile range, and outliers are represented by open circles. Note that only patients with at least 5 weeks of analyzable data were included in this sample. The high-〈*AR*〉 group has high overall ingestion rates while the low-〈*AR*〉 group has a much wider distribution of ingestion rates. The Mann–Whitney *U* test *p*-value between these groups is 0.004. Note that there is significant differentiation between the groups for a range of 〈*AR*〉 threshold values (see “Discussion” section).

For the comparison of activity metrics to ingestion on a treatment-regimen timescale, 46 subjects, with a median overall ingestion rate of 0.88 (IQR = 0.16), had at least 5 weeks of analyzable days, and thus met the previously described data quality criteria. We calculated the mean and standard deviation of both *AR*, i.e., 〈*AR*〉 and *σ*[*AR*], and *rHR*, i.e., 〈*rHR*〉 and *σ*[*rHR*], across all daily values for each patient. Table 2 contains a summary of these mean and standard deviation features. Of these four patient-level features, a relationship to the overall ingestion rate was only seen in 〈*AR*〉. Patients with high 〈*AR*〉 values tended to have high overall ingestion rates; however, patients with lower 〈*AR*〉 values did not necessarily exhibit lower ingestion rates. Figure 5 provides an example of this trend when the patient sample was divided at the median value of 〈*AR*〉 = 0.75. At this cutoff, the subjects with higher 〈*AR*〉 scores had a median ingestion rate of 0.91 (IQR = 0.09) while the subjects with lower 〈*AR*〉 scores had a median ingestion rate of 0.78 (IQR = 0.13), and the Mann–Whitney *U* test indicated that the groups were significantly different (*p* = 0.004). Note that this trend holds and is significant, as determined by the *p*-value of the Mann–Whitney *U* test, for all 〈*AR*〉 cutoff values between 0.58 and 0.86 (see “Discussion” section).

No calculated features other than *AR* and *rHR* (see “Methods” section) were significantly correlated with ingestion on either a regimen-level or daily level.

## Discussion

The DMS utilized in this work provides both objective ingestion records and markers of activity, such as step count and heart rate, in patients with SMI, enabling unique exploration of the relationship between patient behavior and adherence to a treatment regimen. Here, two specific features were developed to characterize patient activity: an activity rhythm score *AR*, which is a measure of the degree to which daily step count patterns are consistent with past behavior, and the relative active-interval heart rate *rHR*, which could be related to the intensity of activity.

At the daily level, we see a direct relationship between daily *AR* and *rHR* and the likelihood of an ingestion on the next day. If these relationships are able to be validated in the future, they would serve as valuable behavioral markers that help to predict and improve ingestion behavior in SMI patients that exhibit poor medication adherence. This improved understanding of a patient’s behavioral state and its link to medication ingestion could provide valuable information to inform clinical decision-making regarding therapy. For example, a disruption in a patient’s activity pattern, particularly when coupled with poor medication adherence, maybe a leading indicator of a worsening clinical state; thus, these metrics could provide early warnings to clinicians prior to more serious outcomes, such as relapse or hospitalization. Although the validated interpretation of the causality of these relationships is beyond the scope of this work, the link between higher daily *AR* and next-day ingestion may suggest that patients with consistent behavioral patterns are more likely to also successfully adhere to a medication regimen. We note that the interpretation of the link between *rHR* and ingestion is somewhat difficult because *rHR* is not a direct measure of activity intensity; it is also dependent on the mean heart rate across the entire day. Thus, a high *rHR* value could be indicative of either a relatively intense active-interval or a day where the patient had a low baseline heart rate. Conversely, a low *rHR* value may not only indicate low-intensity activity but could also be representative of a day in which a patient has an elevated heart rate during inactive periods. Thus, interpreting a causal link would require a more controlled study.

On a patient-regimen level, we found that patients with relatively high mean *AR* values (〈*AR*〉), indicating very consistent activity patterns, were likely to have high overall ingestion rates. The logical inverse, however, was not necessarily true. Patients with lower 〈*AR*〉 had a wide range of overall ingestion rates. We found that 〈*AR*〉 thresholds from 0.58 to 0.86 produced significant differences in the ingestion rate distributions between the relatively high- and low-〈*AR*〉 groups, as measured by the Mann–Whitney *U* test. Cutoff values of 〈*AR*〉 greater than 0.86 did not uniformly produce a significant contrast in ingestion rate because the limited sample size (*N* = 46) led to a small group of relatively high-〈*AR*〉 patients at these cutoffs. The inability of threshold values in 〈*AR*〉 below 0.58 to produce significant contrast is likely due to a lack of correlation between low 〈*AR*〉 values and ingestion rate. While patients with higher 〈*AR*〉 scores all exhibit consistent activity patterns across most of their regimens, patients with low 〈*AR*〉 scores seem to be more behaviorally heterogeneous, which could explain the less robust relationship between low 〈*AR*〉 and ingestion rate.

The uniqueness of the objective ingestion data from this DMS and the activity features developed here represent important evidence of the potential link between patient activity behavior and medication ingestion. However, this work does have several limitations which could be ameliorated by further exploration. First, the ingestion behavior in this data set is skewed towards high ingestion rates, potentially due to a more well-controlled clinical trial setting. Thus, it is difficult to truly determine whether there is a link between any of the activity features and very poor adherence. Future work is needed to apply these algorithms to a larger patient population in a more natural care setting. Some of the data quality requirements for calculating the *AR* feature also further exacerbate the bias of the ingestion data by limiting the data set to patients that had more robust patchwear and thus were inherently more likely to have successfully recorded ingestion events. The utilized criteria for data quality seem to be reasonable, based on our previous work; however, in future work, it would be important to explore the tradeoffs between feature quality and data set bias by tuning the various quality criteria. Additionally, the current activity rhythm methodology is essentially quantifying the similarity of a daily activity pattern to a historical mean pattern that is assumed to be unimodal. The SMI patient population could be prone to a more bimodal distribution of step count patterns; thus, with larger data sets, it could be interesting to characterize the similarity of daily activity to multiple representative patterns. From a data quality standpoint, the use of a 14-s sample of accelerometer data every minute could lead to underestimated step counts if subjects engage in very short bursts of activity. Because this limitation is consistent across all measurements in the data set, and because the algorithm utilizes a temporal resolution of 15 min, it is likely that missed short-duration activity will not significantly affect the 15-min step count values nor the relative patterns of step counts; however, validation with continuous accelerometer data could provide more robust evidence. Finally, this was a retrospective analysis of an available data set, and the study was designed to engineer activity features from the available DMS data. Thus, it will be vital to validate both the observed relationship between these activity metrics and ingestion and the activity rhythm methodology itself using independent data sets. For example, the *AR* score could be compared to markers of circadian rhythm or other techniques for characterizing activity patterns that use different methods for calculating the frequency power spectrum. However, to the best of our knowledge, the publicly available data sets did not have sufficient longitudinal data, i.e., more than 7 days, to validate our methodology. This validation will be a focus of future work.

However, with this data set, we were able to calculate other commonly used non-parametric metrics of rest-activity patterns including interdaily stability (*IS*), intradaily variability (*IV*), and relative activity (*RA*) amplitude calculated using the ten most active daily hours (*M*10) and five least active daily hours (*L*5)^25–27^. The *IV* and *RA* metrics showed modest correlation with the subject-level 〈*AR*〉 metric, with Spearman correlation coefficients of 0.30 and 0.32, respectively; however, none of these rest-activity markers, i.e., *IS*, *IV, RA, M*10, or *L*5, demonstrated any significant correlation with overall ingestion rates. Thus, the *AR* metric, which is dependent only on relative periodicities of the step count pattern and does capture the relationship between activity patterns and medication ingestion, could serve as a valuable new tool to assess the activity behavior of SMI patients. This work represents an important step in leveraging sensor data and the objective ingestion information provided by digital medicine to better characterize patient activity patterns and to explore the behavioral context surrounding medication ingestion.

## Methods

### Digital medicine system (DMS)

The DMS employed here has been fully described in a previous publication^18^. Briefly, the DMS consists of an ingestible sensor embedded in an active pharmaceutical, in this case, the atypical antipsychotic aripiprazole, and a patch attached to the torso that both records ingestion events and contains a three-axis accelerometer and single-lead electrocardiogram (ECG). All collected data is uploaded and placed in cloud storage via a mobile phone application (Fig. 1).

Each measured ingestion is recorded with a timestamp. A recorded ingestion is a robust indicator that an ingestion occurred; however, the lack of an ingestion record could result either from the patient truly not ingesting the medication or from a connectivity issue, e.g., the patient not wearing the patch.

A 14-s sample of the accelerometer data is measured every minute, and an on-board algorithm converts this raw data to a step count, mean acceleration along all three axes, and a mean body orientation angle. The ECG data is collected over a 14-s sample every 5 min, which is concurrent with one of the 14-s accelerometer samples in that period. A mean heart rate is then calculated using the mean of the R-R peak intervals within the 14-s sample.

### Study population

The population analyzed here included 113 subjects across two separately conducted clinical trials^23,24^. All subjects had been diagnosed with schizophrenia, bipolar disorder, or major depressive disorder and were receiving a stable daily dose of aripiprazole prior to the studies. During the trials, patients received the digital version of their aripiprazole regimen for an 8-week period. The studies were reviewed and approved by an appropriate institutional review board (Copernicus Group IRB, Research Triangle Park, NC) and patients were all deemed capable of using the DMS and provided written informed consent. Table 1 contains a demographic and clinical summary of these patients.

### Activity rhythm feature

Our primary aim in this analysis was to probe changes in patient activity patterns across treatment regimens. We chose to use step count as the input data for this analysis, as it should be expected to oscillate approximately daily, with few recorded steps during sleep and a higher step count during waking hours.

To perform this analysis, we first partitioned all patient data into non-overlapping, 15-min intervals and calculated the mean step count within each interval. These 15-min intervals reduced the number of zero-step data points and enabled the use of data quality criteria (see below) based on the availability of both the accelerometer data and the relatively sparse ECG data. For each day, time-series data with a 15-min temporal resolution for that day and the two preceding days were analyzed using a Lomb–Scargle periodogram to determine the characteristic frequency. This three-day window was the shortest timeframe that enabled the robust fitting of the approximately one-day periodicity of the time-series data. The Lomb–Scargle periodogram is an algorithm for performing power spectrum analysis for unevenly sampled time-series data^20–22^. It is thus ideally suited to the current application, in which missing accelerometer data is common due to imperfect patchwear^28^. Indeed, the Lomb–Scargle periodogram has been previously applied for identifying frequencies in other biological time-series^29–31^. The equation for the Lomb–Scargle periodogram, which was implemented in Python via the *scipy* package^32^, can be written as follows:

3 $$P\left( f \right) = \frac{1}{2}\left[ {\frac{{\left( {\mathop {\sum}\nolimits_n {s_n{\mathrm{cos}}\left( {2\pi f\left[ {t_n - \tau } \right]} \right)} } \right)^2}}{{\mathop {\sum}\nolimits_n {{\mathrm{cos}}^2\left( {2\pi f\left[ {t_n - \tau } \right]} \right)} }} + \frac{{\left( {\mathop {\sum}\nolimits_n {s_n{\mathrm{sin}}\left( {2\pi f\left[ {t_n - \tau } \right]} \right)} } \right)^2}}{{\mathop {\sum}\nolimits_n {{\mathrm{sin}}^2\left( {2\pi f\left[ {t_n - \tau } \right]} \right)} }}} \right],$$

where

4 $$\tau = \frac{1}{{4\pi f}}{\mathrm{tan}}^{ - 1}\left( {\frac{{\mathop {\sum }\nolimits_n {\mathrm{sin}}\left( {4\pi ft_n} \right)}}{{\mathop {\sum }\nolimits_n {\mathrm{cos}}\left( {4\pi ft_n} \right)}}} \right).$$

Here, *t*_*n*_ is the *n*th measurement time-point, *s*_*n*_ is the step count at the *n*th time-point, and *f* is the frequency for which the periodogram amplitude *P* is being calculated. The characteristic frequency *f*_c_ for a given day is then defined as the frequency which maximizes the Lomb–Scargle periodogram for the three-day window leading up to and including that day. For instance, if the step-count pattern repeated in a perfect daily rhythm, *f*_c_ would be equal to 1⁄(1 *day*). Here, we are interested in determining if a patient’s step count pattern on a given day is consistent with that patient’s previous activity rhythm. As such, we compare each day’s *f*_c_ to the mean frequency across all previous days for that patient in order to define the activity rhythm score *AR*, such that

5 $$AR_m = \frac{{P_m\left( {\left\langle f \right\rangle } \right)}}{{P_m\left( {f_c^m} \right)}},$$

where *AR*_*m*_ is the activity rhythm score on the *m*th day, $f_{\mathrm{c}}^m$ is the characteristic frequency on the *m*th day, *P*_*m*_ is the Lomb–Scargle periodogram amplitude calculated using the 3-day window of step count data ending on the *m*th day, and 〈*f*〉 is the mean of the characteristic frequencies of all previous days for that patient (Fig. 2). Since $P_m\left( {f_{\mathrm{c}}^m} \right)$ is, by definition, the maximum value of *P*_*m*_, *AR*_*m*_ exists on the range (0,1]. A value of *AR*_*m*_ = 1 indicates that $f_{\mathrm{c}}^m = \left\langle f \right\rangle$ while a value of *AR*_*m*_ approaching 0 indicates that oscillation at the historic mean frequency 〈*f*〉 is not present in the 3-day window ending on the *m*th day. Thus, larger values of *AR*_*m*_ indicate that the *m*th day step count pattern is more consistent with previous days’ activity rhythms.

To ensure data quality, 15-min intervals with fewer than 10 accelerometer records or fewer than 2 ECG records were excluded, and periodogram spectra were not calculated for 3-day windows with fewer than two-thirds of the expected 15-min-interval time-points, i.e., 192 time-points (96 windows/day). Other data quality criteria, e.g., requiring one-half or three-fourths of the expected time-points, were explored and did not significantly alter any findings. Additionally, we required that there be at least 5 baseline power spectra in order to calculate 〈*f*〉 to ensure a reasonably stable baseline value for 〈*f*〉; thus, the eighth day of a treatment regimen is the first day for which a patient could receive an *AR* score.

### Active heart-rate feature

In order to glean insight into the intensity of activity undertaken by patients, we first identified active intervals for each patient-day. This active-interval designation was again performed with a minimum temporal resolution of 15-min. Here, a 15-min interval was identified as “active” if at least one-third of the available accelerometer records within the 15-min interval reported a vertical posture angle, i.e., an angle greater than or equal to 30° above horizontal, and a step count greater than 0 (Fig. 3a). We then calculated an active-interval relative heart rate *rHR*_*A*_, such that

6 $$rHR_m = \frac{{\left\langle {Active\,HR} \right\rangle _m}}{{\left\langle {HR} \right\rangle _m}},$$

where *rHR*_*m*_ is the patient’s active-interval relative heart rate on the *m*th day, $\left\langle {Active\,HR} \right\rangle _m$ is the average heart rate within all active intervals on the *m*th day, and 〈*HR*〉_*m*_ is the patient’s average heart rate across the entirety of the *m*th day (Fig. 3b). Thus, if the patient has a higher than average heart rate during his or her active intervals, *rHR*_*m*_ would be >1 for that day.

We also calculated daily mean step count, daily mean heart rate, daily heart rate standard deviation, daily number of active intervals, and a heart rate analog of the activity rhythm score.

### Comparison of activity features and ingestion

We then compared these activity features to medication ingestion on both a daily basis and on a treatment-regimen timescale. To explore the relationship between daily activity and ingestion and investigate the feasibility of predicting ingestions based on behavioral patterns, we compared a patient’s *m*th-day activity, i.e., *AR*_*m*_ and *rHR*_*m*_, to that patient’s medication ingestion on day *m* + 1. For this daily ingestion, the presence of recorded ingestion on day *m* + 1 was considered a dosing success, and the lack of ingestion on day *m* + 1 was considered a dosing failure, provided that day *m* + 1 contained at least two-thirds of the expected accelerometer records for that day. Days that contained fewer than this number of accelerometer records were excluded from the daily analysis. This was done to increase our confidence that a lack of a recorded ingestion was actually indicative of a missed ingestion and not just a result of a lack of patchwear or other connectivity issues. We chose two-thirds as the threshold for required accelerometer records as a compromise between maximizing the number of analyzable days and maximizing our confidence in the day representing a truly missed ingestion. Other threshold values were explored and did not significantly alter our results.

For the regimen-level analysis, we compared the mean and standard deviation of each patient’s *AR* and *rHR* values across all days to that patient’s overall ingestion rate, defined as the number of days during which an ingested dose was recorded divided by the expected number of ingested doses across the entire regimen, which is defined as the time period between the first and last recorded patch records. Only subjects that had at least five weeks of analyzable data, including the baseline period, were included in this analysis. This cutoff was chosen to ensure that patient-level ingestion rates were not overly sensitive to single data points. This comparison provides insight into whether patients’ overall ingestion patterns are associated with the consistency of their activity patterns over the full treatment regimen. The significance of the difference in the ingestion rates between patients with high and low 〈*AR*〉 was determined using the Mann–Whitney *U* test.

All analysis was performed in Python 3.7.

### Reporting summary

Further information on research design is available in the Nature Research Reporting Summary linked to this article.

## Supplementary information

Reporting Summary

## Data Availability

Based on the proprietary nature of the data, it may not be made available for a period of at least 5 years from publication. All requests would require evaluation on an individual basis and can be made by contacting jeffrey.cochran@otsuka-us.com or OtsukaUS@druginfo.com. The authors made the appropriate materials available to the editorial staff during the review process for verification of results.

## Code availability

The code described here was written using Python 3.7. Based on the proprietary nature of the algorithms, the code may not be made available for a period of at least 5 years from publication. All requests would require evaluation on an individual basis and can be made by contacting jeffrey.cochran@otsuka-us.com or OtsukaUS@druginfo.com. The authors made the appropriate materials available to the editorial staff during the review process for verification of results.
