# Supplementary material for: Characterization of activity behavior using a digital medicine system and comparison to medication ingestion in patients with serious mental illness
Source: NPJ Digit Med. 2021 Apr 6;4:63. doi: 10.1038/s41746-021-00436-1 (PMC8024287; doi:10.1038/s41746-021-00436-1)
Supplement: Supplementary file 1 — Reporting Summary [file 41746_2021_436_MOESM1_ESM.pdf]

nature research

Corresponding author(s): Jeffrey M Cochran

Last updated by author(s): Mar 1, 2021

Reporting Summary

Nature Research wishes to improve the reproducibility of the work that we publish. This form provides structure for consistency and transparency in reporting. For further information on Nature Research policies, see our Editorial Policies and the Editorial Policy Checklist.

Statistics

For all statistical analyses, confirm that the following items are present in the figure legend, table legend, main text, or Methods section.

n/a	Confirmed	
☐	☒	The exact sample size (n) for each experimental group/condition, given as a discrete number and unit of measurement
☐	☒	A statement on whether measurements were taken from distinct samples or whether the same sample was measured repeatedly
☐	☒	The statistical test(s) used AND whether they are one- or two-sided<br>*Only common tests should be described solely by name; describe more complex techniques in the Methods section.*
☐	☒	A description of all covariates tested
☐	☒	A description of any assumptions or corrections, such as tests of normality and adjustment for multiple comparisons
☐	☒	A full description of the statistical parameters including central tendency (e.g. means) or other basic estimates (e.g. regression coefficient) AND variation (e.g. standard deviation) or associated estimates of uncertainty (e.g. confidence intervals)
☐	☒	For null hypothesis testing, the test statistic (e.g. F, t, r) with confidence intervals, effect sizes, degrees of freedom and P value noted<br>*Give P values as exact values whenever suitable.*
☒	☐	For Bayesian analysis, information on the choice of priors and Markov chain Monte Carlo settings
☒	☐	For hierarchical and complex designs, identification of the appropriate level for tests and full reporting of outcomes
☒	☐	Estimates of effect sizes (e.g. Cohen's d, Pearson's r), indicating how they were calculated

Our web collection on statistics for biologists contains articles on many of the points above.

Software and code

Policy information about availability of computer code

Data collection	Clinical data was collected via clinical trials using SAS. Proprietary firmware algorithms were used for data processing, and Python 3.7 was used for collection and cleaning of all processed sensor data.
Data analysis	All data analysis was performed in Python 3.7. The development of the algorithms described in the manuscript was a primary purpose of this research. Due to the proprietary nature of the code, we request that data not be made publicly available for a period of 5 years and then only based on a review of individual requests.

For manuscripts utilizing custom algorithms or software that are central to the research but not yet described in published literature, software must be made available to editors and reviewers. We strongly encourage code deposition in a community repository (e.g. GitHub). See the Nature Research guidelines for submitting code & software for further information.

Data

Policy information about availability of data

All manuscripts must include a data availability statement. This statement should provide the following information, where applicable:

- Accession codes, unique identifiers, or web links for publicly available datasets
- A list of figures that have associated raw data
- A description of any restrictions on data availability

Based on the proprietary nature of the data, it may not be made available for a period of at least 5 years from publication. Requests would require evaluation on an individual basis. The authors made the appropriate materials available to the editorial staff during the review process for verification of results.

Field-specific reporting

Please select the one below that is the best fit for your research. If you are not sure, read the appropriate sections before making your selection.

☐ Life sciences ☒ Behavioural & social sciences ☐ Ecological, evolutionary & environmental sciences

For a reference copy of the document with all sections, see nature.com/documents/nr-reporting-summary-flat.pdf

Behavioural & social sciences study design

All studies must disclose on these points even when the disclosure is negative.

Study description	This is a quantitative, retrospective analysis designed to develop novel behavior markers and explore potential correlations with measured medication ingestion.
Research sample	All subjects were recruited as part of two clinical trials for a digital medicine system, and all had been previously diagnosed with schizophrenia, bipolar I disorder, or major depressive disorder. This group was chosen because those are the three approved indications for the active pharmaceutical product used in the digital medicine system.
Sampling strategy	Sample size calculations were determined individually for each trial to ensure the necessary statistical power to assess each trial's primary and secondary endpoints. This retrospective analysis opportunistically utilized the previously collected trial data and thus was not statistically powered to a specific sample size.
Data collection	All clinical data was collected by clinical trial personnel at clinical sites. Sensor data, including accelerometer, ECG, and ingestion records, were longitudinally collected via the patch component of the digital medicine system. This data collection requires that the patient apply and wear the patch but does not require interaction with study personnel.
Timing	Data from one clinical trial was collected from August 2014 to July 2015. Data from the other clinical trial was collected between March 2016 and September 2016.
Data exclusions	All subjects were included in the initial analysis. Because longitudinal analysis was performed, individual days were excluded due to insufficient data on those days (e.g., less than 2/3 of expected data over a 3-day period). In some cases, this caused some subjects to have insufficient data to apply the behavioral algorithms (e.g., fewer days than required for baseline window). All exclusion criteria and the number of remaining subjects are delineated in the manuscript.
Non-participation	For one of the trials, 11 of the 49 enrolled subjects did not complete the study for any reason. In the other trial, 18 of the 67 enrolled subjects did not complete the study for any reason.
Randomization	Subjects were not randomized into separate groups.

Reporting for specific materials, systems and methods

We require information from authors about some types of materials, experimental systems and methods used in many studies. Here, indicate whether each material, system or method listed is relevant to your study. If you are not sure if a list item applies to your research, read the appropriate section before selecting a response.

Materials & experimental systems

n/a	Involved in the study	
☒	☐	Antibodies
☒	☐	Eukaryotic cell lines
☒	☐	Palaeontology and archaeology
☒	☐	Animals and other organisms
☐	☒	Human research participants
☐	☒	Clinical data
☒	☐	Dual use research of concern

Methods

n/a	Involved in the study	
☒	☐	ChIP-seq
☒	☐	Flow cytometry
☒	☐	MRI-based neuroimaging

Human research participants

Policy information about studies involving human research participants

Population characteristics	See above
Recruitment	Patients were recruited by individual clinical trial sites.
Ethics oversight	Copernicus Group IRB, Research Triangle Park, NC

Note that full information on the approval of the study protocol must also be provided in the manuscript.

Clinical data

Policy information about clinical studies

All manuscripts should comply with the ICMJE guidelines for publication of clinical research and a completed CONSORT checklist must be included with all submissions.

Clinical trial registration	NCT02219009, NCT02722967
Study protocol	clinicaltrials.gov
Data collection	Data from one clinical trial was collected from August 2014 to July 2015 at six trial sites (Cerritos, California, United States, 90703; Garden Grove, California, United States, 92845; Oceanside, California, United States, 92056; Atlanta, Georgia, United States, 30331; Chicago, Illinois, United States, 60640; Saint Louis, Missouri, United States, 63141). Data from the other clinical trial was collected between March 2016 and September 2016 at four clinical trial sites (Granada Hills, California, United States, 91344; Rochester, New York, United States, 14603; Durham, North Carolina, United States, 27714; Richmond, Virginia, United States, 23230).
Outcomes	Primary and secondary endpoints were defined for each clinical trial. This work, however, did not require or utilize any primary or secondary endpoints due to its retrospective and exploratory nature.
